# An Ear Wearable Device System for Facial Emotion Recognition Disorders

**DOI:** 10.3389/fbioe.2021.703048

**Published:** 2021-06-23

**Authors:** Zhengxu Lian, Yingjie Guo, Xinyu Cao, Wendi Li

**Affiliations:** ^1^School of Medicine and Bioinformatics Engineering, Northeastern University, Shenyang, China; ^2^Software College of Northeastern University, Shenyang, China

**Keywords:** facial emotion recognition, pressure recognition, multi-modal physiological signal, wearable device, facial emotion recognition disorder

## Abstract

A wearable device system was proposed in the present work to address the problem of facial emotion recognition disorders. The proposed system could comprehensively analyze the user’s own stress status, emotions of people around, and the surrounding environment. The system consists of a multi-dimensional physiological signals acquisition module, an image acquisition and transmission module, a user interface of the user mobile terminal, and a cloud database for data storage. Moreover, a deep learning based multi-model physiological signal pressure recognition algorithm and a facial emotion recognition algorithm were designed and implemented in the system. Some publicly available data sets were used to test the two algorithms, and the experiment results showed that the two algorithms could well realize the expected functions of the system.

## Introduction

As an important way of emotional expression and cognition, facial expressions are an indispensable part of our daily activities. Being a form of one’s response to happenings in the objective world, emotions play an important role in people’s real life and spiritual life. Obstacles in recognition of facial emotions will inevitably lead to problems in interpersonal communication. Shen et al. (2015) have found that the mechanisms of facial emotions recognition are complex and are not the functions of one single area in the brain; instead, different loops are formed between different regions, and damages to these loops would lead to facial emotion recognition disorders, manifested by such diseases as schizophrenia, cerebrovascular accidents, dementia syndrome, Parkinson’s disease, depression, autism, epilepsy, traumatic brain injury, and multiple sclerosis. Therefore, early detection and identification of potential facial emotion recognition disorders will facilitate the judgment, treatment and community management of nervous system diseases.

At the same time, with the rapid development of the wearable technology in recent years, wearable devices have provided a popular solution and played an important role in health monitoring, safety monitoring, family rehabilitation, efficacy evaluation, early detection of diseases, and other related fields (Jia et al., 2017). The purpose of this research is to design a wearable device system for facial emotion recognition disorders, and the system could comprehensively analyze the user’s own psychological stress, other people’s emotions and surrounding environment, thus contributing to the prevention, monitoring, and management of related diseases.

This system is expected to provide a solution to the following fields. (1) It can be used as a monitoring system for early diagnosis, prevention and treatment of diseases related to facial emotion recognition, such as anxiety, to prevent it from escalating into more serious diseases like depression. (2) The system can provide a technical basis for treatment of specific diseases. For example, in the case of patients with autism who are sensitive to mathematical laws, the system can transform the emotional data of others into more regular games to help autistic patients better recover and recognize their emotional recognition capacity. (3) The system can also be used as a data collection platform to provide more sample data for the research on neurological diseases.

Wearable devices for monitoring of users’ physiological signals and detection of psychological pressure have drawn wide attention from researchers around the world. Such signals come from all parts of the body, such as Electrocardiograph (ECG), Electro- myogram (EMG), Electroencephalogram (EEG), and respiration. A new wearable ECG monitoring system based on active cables and smart electrodes developed by the KTH Royal Institute of Technology includes a hand-held personal health assistant, an active cable and 10 smartelectrodes, which are attached to specific parts of the patient’s body from chest to calf, and can obtain high-quality ECG data (Yang et al., 2008). In a study by Hasanbasic et al. (2019), they monitored students’ ECG and skin electrical activity signals by wearable sensors in real time, and classified by machine learning algorithms such as SVM to identify the students’ stress level in a specific environment such as during exams (Hasanbasic et al., 2019). Montesinos et al. (2019) employed multi-modal machine learning and sensor fusion technology to detect the occurrence of acute stress events.

In these previous publications, researchers employed wearable sensors to collect physiological signals, discriminated and classified psychological pressure through machine learning. However, physiological-signal monitoring devices mentioned in the above studies are expensive and cumbersome. As a result, ordinary users often find it hard to afford these expensive devices and inconvenient to wear these devices in daily life, which makes it impossible to popularize these devices. Moreover, the previous studies have failed to measure the surrounding environment when stress occurs, and hence could not assess the impact of the surrounding environment on facial emotion recognition disorders.

Machine learning models, especially deep learning algorithms, are popular solutions to classification of physiological information and it is assumed that these models could bring new breakthroughs to facial emotion recognition disorders. In fact, research on facial emotion recognition based on deep learning has been relatively mature. Arriaga et al. (2017) proposed a lightweight convolutional neural network, which reached an emotion recognition accuracy close to human on the FER2013 database. Therefore, it is feasible to achieve the goal of recognizing emotions in interpersonal scenarios by improving machine learning models.

The present work proposes a wearable device system that is cost effective and can keep track of changes in the surrounding environment. The system achieves the expected functions by a self-designed multi-modal psychological signal-based stress recognition deep learning algorithm and an improved facial emotion recognition algorithm.

The software of the proposed system was designed as follows. A multi-modal psychological signal-based stress recognition multi-head Convolutional Neural Networks (CNN) model was designed, the facial emotion recognition model based on the mini_Xception CNN was improved; and the algorithms were deployed on the cloud server; A cloud database was constructed to store the physiological signals and analysis results obtained by the above-mentioned algorithms, and a mobile interface for user interaction was developed. The hardware of the system was developed as follows. An ergonomic ear wearable device was designed, which comprised of sensors for the photoelectric volumetric heart rate, triaxial acceleration, skin electricity and body temperature, cameras used for collection of the facial images of the people whom users interact with and the surrounding environment, and a Wi-Fi module for data transfer with the cloud server.

## Materials and Methods

### Overall Framework of Wearable Device System

**FIGURE 1:**
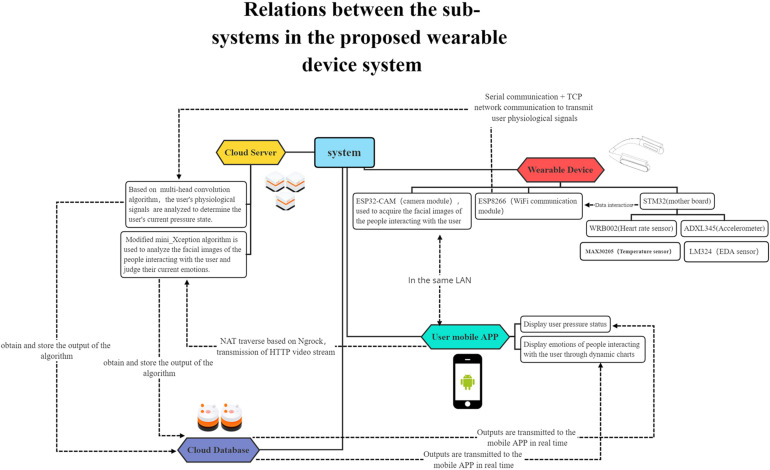
Relations between the sub-systems in the proposed wearable device system.

This system is composed of five sub-systems: an image acquisition and transmission system, a human physiological signal collection and transmission system, an algorithm analysis system deployed on cloud server, a cloud database and an user mobile APP, as shown in Figure 1. (1) The image acquisition and transmission system realizes real-time image acquisition through cameras, and transmits videos to the cloud server via intranet transparent transmission technology through module integrated WI-FI chips, thus completing wireless image transmission. (2) In the human physiological signal acquisition and transmission system, small-sized sensors are used to make the device portable and easy to use; the physiological signals collected by the sensors are input into the ESP8266 WI-FI communication module connected with the serial communication ports through the main development board, and the module further uploads the data to the cloud server using the Transmission Control Protocol (TCP) transmission protocol. (3) On the cloud server, a multi-modal physiological signal-based stress recognition model and a facial emotion recognition model are deployed to acquire and analyze data, and provide feedback to the cloud database. (4) The cloud database records the collects raw data and algorithm analysis results, and returns the user stress state and the facial emotion prediction result of people around to the user through the mobile APP. (5) The mobile APP receives the algorithm analysis results returned by the cloud database and displays them on the user interface for users to view.

### Hardware Circuit and Appearance Design

**FIGURE 2:**
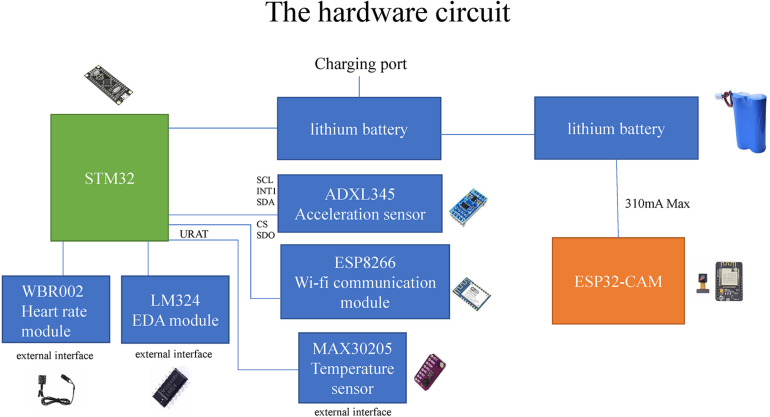
The hardware circuit.

In the hardware circuit design, the image acquisition and transmission system and the human physiological signal acquisition system are independent of each other. As shown in Figure 2, the image acquisition and transmission system is composed of the ESP32CAM module with integrated cameras and WI-FI chips powered by lithium batteries. In the human physiological signal acquisition system, STM32 is used as the main development board, and ADXL345 acceleration sensor, LM324 skin electrical sensor, MAX30205 body temperature sensor and photoelectric volumetric WRB002 heart rate sensor are connected to the corresponding serial ports of the development board, and are also powered by lithium batteries. The serial port data exchange end of the motherboard is connected to the WI-FI module ESP8266, through which physiological signals can be transmitted to the cloud server.

**FIGURE 3:**
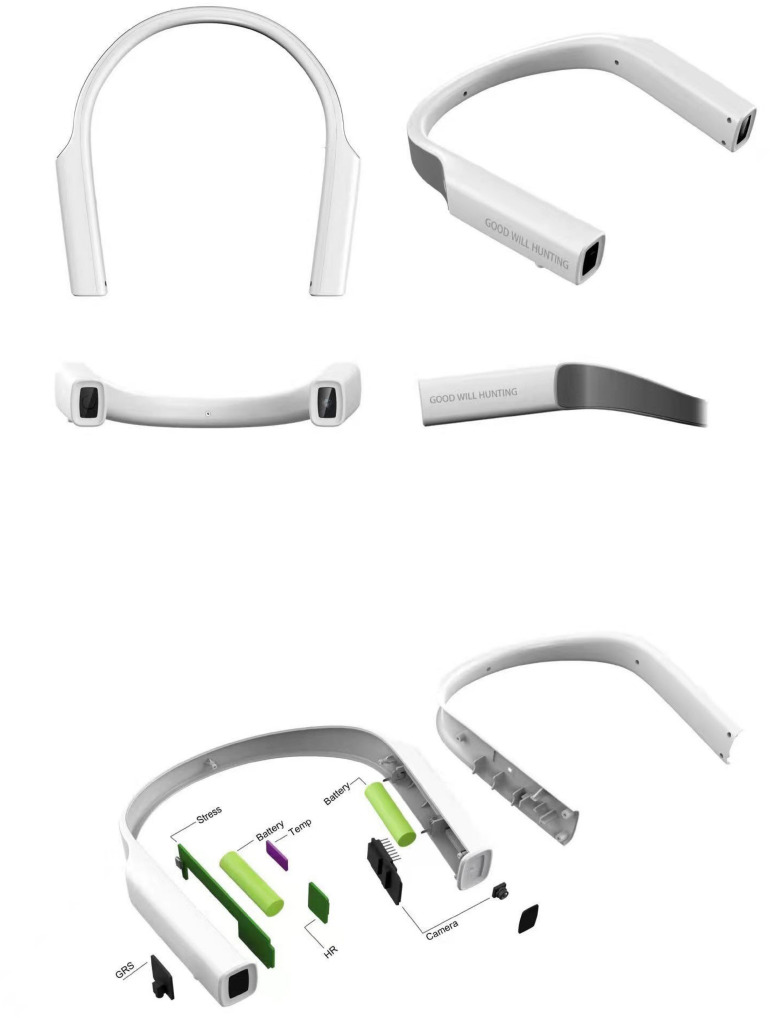
Wearable device appearance and internal structure.

The wearable device presents a U-shaped elastic ring structure, which is placed above the ear and close to the head. Each sensor opening is arranged on the side to facilitate physiological signal acquisition. U-shaped front-end cameras can collect the facial images of the people whom users interact with and the surrounding environment. Figure 3 presents the appearance and internal structure of the device.

### Software Design

**FIGURE 4:**
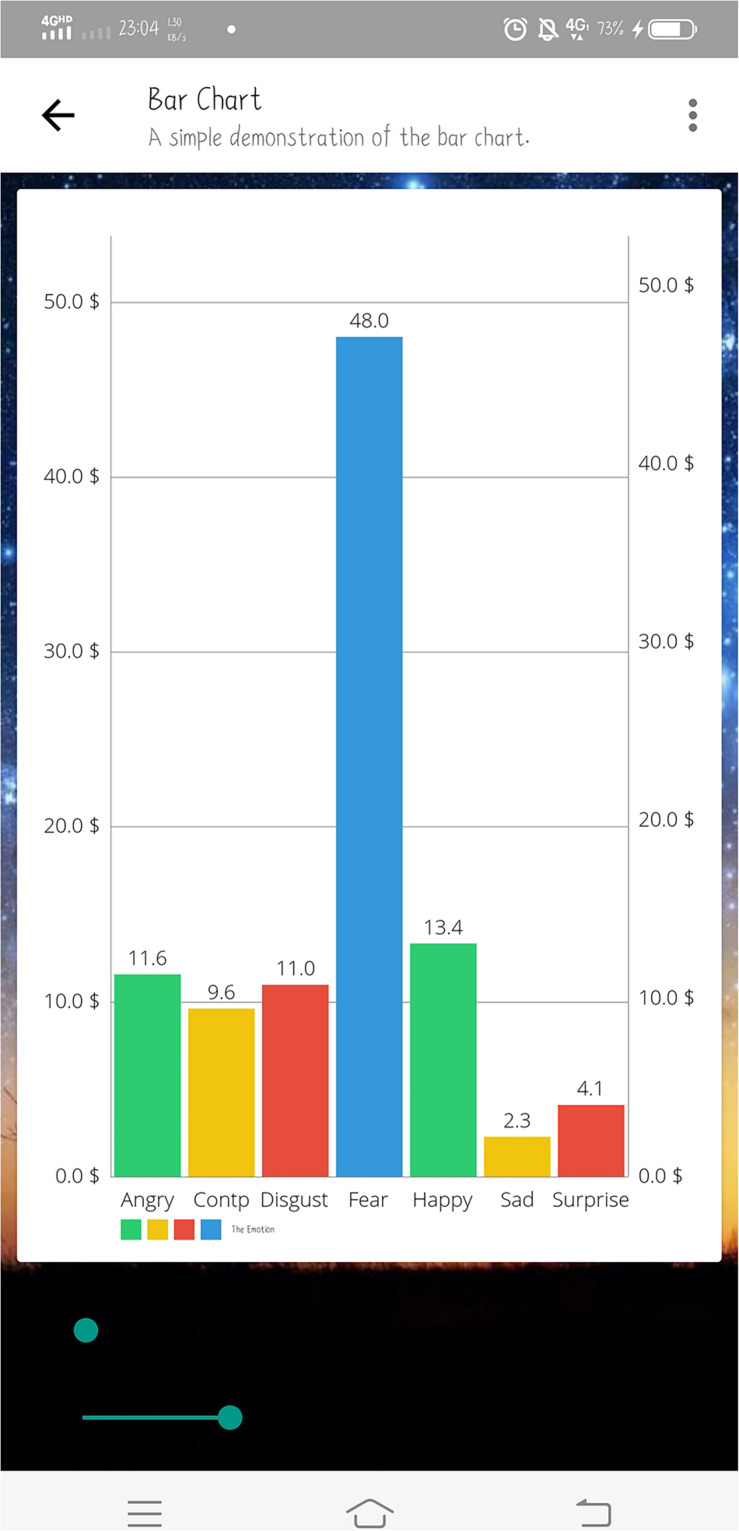
Display of analysis results on the mobile app.

The experimental software includes a mobile APP, a cloud database and algorithm analysis system deployed on the cloud server. The mobile APP interface is written in Java and can be used on Android mobile devices. By receiving the algorithm analysis results returned from the cloud database, the app presents the user’s psychological stress status and the facial emotion recognition results of people around in real time (Figure 4). The cloud database uses MySQL language to record and store the raw physiological signals from sensors and algorithm analysis results. In order to facilitate the follow-up studies about the impacts of the scenes on the user’s stress state, the cloud database will also store the images collected by the camera module when the algorithm judges that the user is under stress. The cloud server adopts the Windows Server 2012 system. The working principles of the multi-modal physiological signal-based stress recognition model and the facial emotion recognition model deployed on the cloud server will be discussed in detail in Sections “Facial Emotion Recognition Algorithm Description” and “Multi-modal Physiological Signal-based Stress Recognition Algorithm.”

### Facial Emotion Recognition Algorithm Description

#### Image Texture Feature Extraction

Texture, a common feature of images, has been widely used in various image segmentation, classification and recognition tasks. Tyagi (2018) points out that there is no universally-accepted definition for the image texture at present. However, in some studies (Smith and Chang, 1996; Hall-Beyer, 2007), texture is defined as the surface roughness and roughness of objects, which are unique and show certain patterns. Texture features are reflected by gray intensity distribution pattern in images, and these features have been described in diverse forms in the field of digital image processing. Khaldi et al. (2019) points out that gray level co-occurrence matrix (GLCM) is often considered as an accurate method of texture feature extraction, and has seen wide adoption in various fields because of its simplicity and high efficiency. For an image I of the size of *N* × *M*, with a given displacement vector (Δ*x*, Δ*y*), the gray level co-occurrence matrix M can be obtained by Eq. 1.

(1) $$M\text{(}p\text{,}q\text{)=}\sum_{i=1}^{N}\sum_{j=1}^{M}\{\begin{array}{c} 1\\ 0 \end{array}\begin{array}{c} \text{if}I(i,j)=p\;and\\ I(i+\Delta x,j+\Delta y)=q\\ \text{otherwise} \end{array}$$

Usually, some statistical indicators are used to characterize the gray level co-occurrence matrix and hence reflect that texture features of images. In the present work, the following statistical indicators were employed to calculate the eigenvalue of the local gray level co-occurrence matrix of the images: the mean, standard deviation (std), contrast, dissimilarity, homogeneity, angular second moment (ASM), energy, maximum and entropy, so as to generate images that reflect the texture features of the image.

**FIGURE 5:**
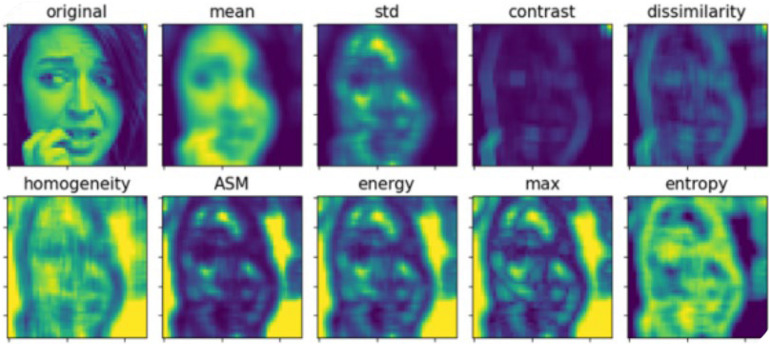
Grayscale pseudo-color images for nine features.

Figure 5 shows the pseudo-color images of a human face after extraction of texture features. An image with a size of 48 × 48 and a gray scale range of 0–255 is used for testing. The lighter colors corresponds to the higher gray values. The pseudo-color images clearly presents the texture features extracted by the gray level co-occurrence matrix based on different statistical indicators.

#### Algorithm Description

To solve the problem of parameter redundancy, improve the model’s generalization capacity, and reduce the processing burden of the hardware, Arriaga et al. (2017) proposed a lightweight CNN: mini_Xception. This network borrowed the ideas of deep segmentable volume and residual network from the Google mainstream CNN Xception (Chollet, 2017), to achieve higher accuracy under small model complexity.

**FIGURE 6:**
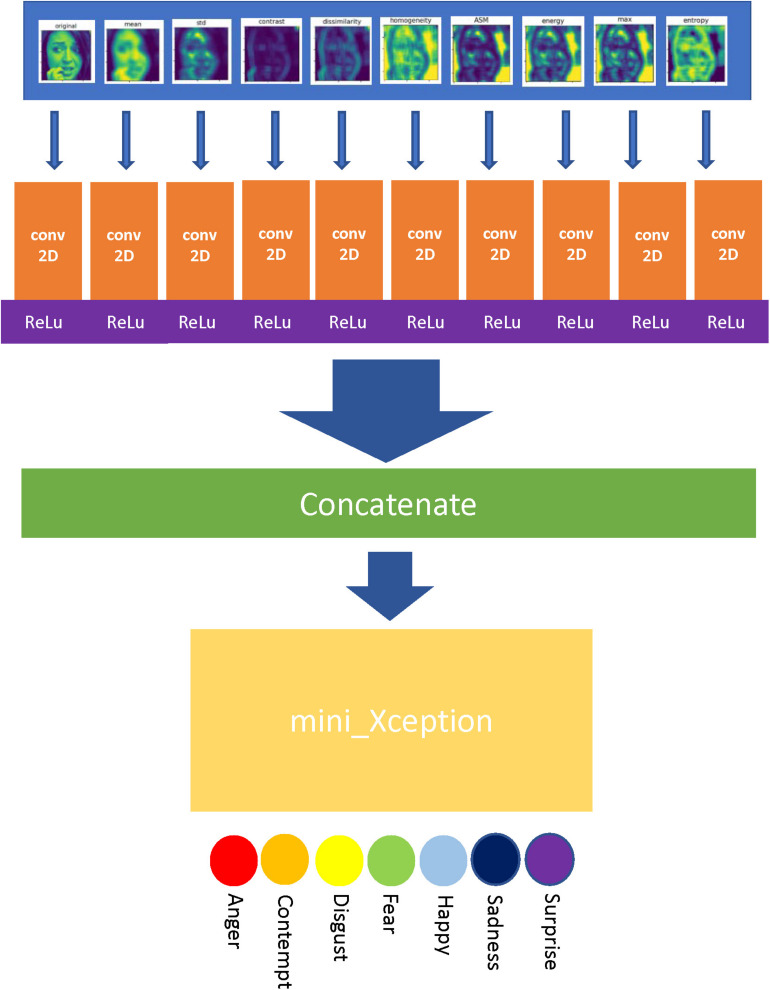
Algorithm implementation flow chart.

In order to improve the performance of mini_Xception in facial emotion recognition task, the gray level co-occurrence matrix was introduced in our method to extract texture features from the input images so as to enrich the types and scale of data for the model. The algorithm implementation flow is shown in Figure 6.

As Figure 6 shows, the algorithm first extracts the texture features of the input image to generate nine texture-feature images. After that, ten images including the original image are input into the algorithm through 10 separate channels, and are filtered by 3 × 3 convolution networks with the 16 dimensions. After passing the activation layer of the Rectified Linear Unit (ReLu) function, the features of each channel are fused and input into the mini_Xception model. At last, the mini_Xception model outputs the probabilities of seven predicted emotions through the softmax activation layer, and takes the emotion with the largest probability as the final predition outcome. The Adam optimizer is used as the weight optimizer of the algorithm network in the training process.

### Multi-Modal Physiological Signal-Based Stress Recognition Algorithm

#### Batch Standardization

Firstly, the physiological signals of the input model are standardized in batches. The mean and variance of a batch of data can be calculated by Eqs 2 and 3, respectively, and then each indicator in the batch of data is standardized by Eq. 4, and finally the weight of the data is corrected to achieve the result. By standardizing the variance of the training set data, the values of feature vectors in each dimension are treated equivalently, and are made to follow the normal distribution with the mean value of 0 and the variance of 1. Thus the problem of unbalanced weight caused by the difference in the values of feature vectors can be avoided (Tang, 2017).

(2) $$\mu_{B}=\frac{1}{m}\,\sum_{i=1}^{m}x_{i}$$

(3) $$\sigma_{B}^{2}=\frac{1}{m}\,\sum_{i=1}^{m}{\left(x_{i}-\mu_{B}\right)}^{2}$$

(4) $${\hat{x}}_{i}=\frac{x_{i}-\mu_{B}}{\sqrt{\sigma_{B}^{2}+\text{ε}}}$$

#### Slicing

Usually, Eq. 5 is used to transform single-row single-dimension inputs into multi-row and multi-dimension. In Eq. 5, “input” is the input value of the model, “slice” is the slice length, and *a*_*k*_ is a row of vector of the original data. The purpose of this method is to change the input from single time point to multi-points time period, and to provide the data with continuous physiological information. Because of the large individual differences of one-dimensional physiological data, it has poor robustness in model adaptation. In addition, the separate time points of physical signals are not all index data, and cannot be used to indicate a certain physiological state just because the value of which reach a specific threshold. Therefore, the input of multi-dimensional time period data is needed to ensure the introduction of continuous characteristics of signals, so as to make the model more robust and practical (Jia et al., 2017).

(5) $$\text{Input}=\left(a1,a2,a3\ldots a_{k}\right)T\left(k=1,2,3\ldots \text{Slice}\right)$$

#### Model Design

In the model proposed in this study, the multi-channel concatenate layer fusion model was used for training.

The main working principle is shown in Eq. 6.

(6) $$\text{Zconact}=\sum_{i=1}^{c}X\,i*K\,i+\sum_{i=1}^{c}X\,i*K\,i+c+\cdots$$

**FIGURE 7:**
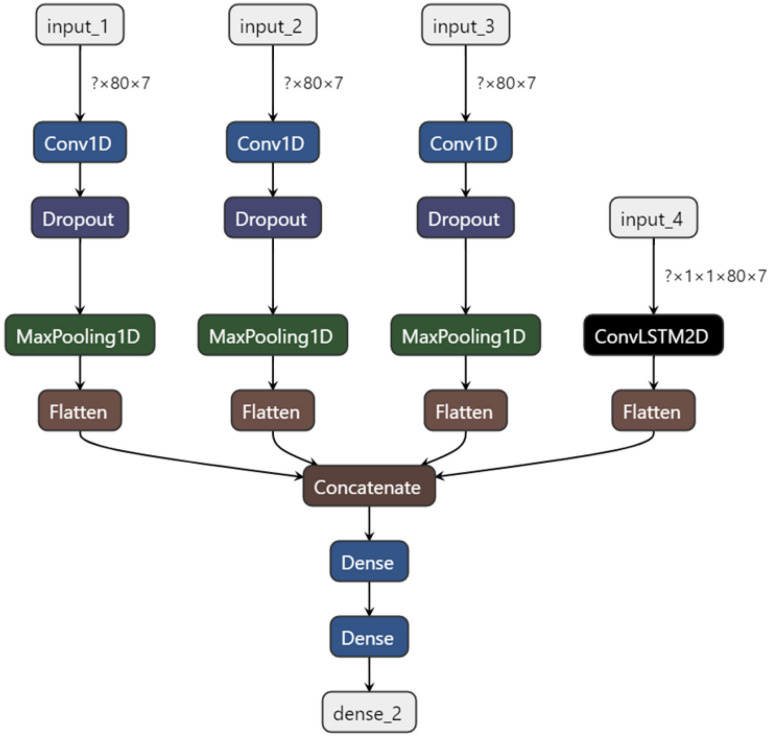
Model structure.

The model structure is shown in Figure 7.

As shown in Figure 7, there are four basic input channels of the model in this experiment. The first three channels share the same workflow, all consisting of an input layer, a one-dimensional convolution layer, a random inactivation layer, a maximum pooling layer and a flattening layer. The convolution kernel size was set at 3, 5, and 11 to extract features from different scales. The fourth channel is composed of a Convolution+Long Short-Term Memory-2D (ConvLSTM-2D) model and a flatten layer. Among them, the traditional two-stream LSTM model cannot only improve the performance of the neural network by making better use of the dependency product between sequence frame data (Jie et al., 2021), but also introduce the long-term and short-term memory mechanism to the model. Through the convolution layer, the relationship between time series can be obtained, and at the same time, the spatial features can be extracted, and thus the spatio-temporal features are obtained. Therefore, the two-stream convolution of LSTM combined with Attention-Conv can better analyze the spatio-temporal relationship of local features. Then, the data from the four input channels are weighted and fused, and the feature dimension is reduced through the full connection layer, after which four kinds of stress recognition results are output: physiological stress, cognitive stress, emotional stress and relaxed state.

### Evaluation Indicators

To evaluate the experimental results, the following evaluation criteria are defined and used.

#### Loss Function

In multi-classification problems, the cross entropy loss functions of Eqs 7 and 8 are often used, where *y* is the predicted value, and *y_hat* is the label value. By continuously reducing the value of the loss function *L* to 0, the predicted result and the actual label value could be matched.

(7) $$\text{Soft }\max\left(y_{\text{i}}\right)=\frac{e^{y_{i}}}{\sum_{i=1}^{n}e^{y_{i}}}$$

(8) $$L\,\left(y,y_{-}\,h\,a\,t\right)=-\frac{1}{n}\,\sum_{i=1}^{n}y_{-}\,h\,a\,t_{i}\times \log\left(\text{Soft }\,\max\left(y_{i}\right)\right)$$

#### Accuracy

In evaluation of multi-classification tasks, multi-classification problems are often transformed into multiple two-classification problems. The selected class is set as positive (P), while the rest is set as negative (N). If the prediction result matches the label, the classified target is marked by a prefix T; otherwise, it is marked by the prefix F.

Equation 9 shows the calculation of the classification accuracy, which represents the ratio of correct prediction times to all prediction times.

(9) $$\text{Accuracy}=\frac{\text{TP}+\text{TN}}{\text{TP}+\text{TN}+\text{FP}+\text{FN}}$$

#### Evaluation Indicators of Test Set

Equation 10 is used to calculate the classification precision of a random class *i*, which describes the proportion of true positives in samples predicted to be positives.

(10) $${\text{Precision}}_{i}=\frac{{\text{TP}}_{i}}{{\text{TP}}_{i}+{\text{FP}}_{i}}$$

Equation 11 is the recall rate of classification of Class *i*, which represents the number of samples predicted to be positive among the samples that are really positive.

(11) $${\text{Recall}}_{i}=\frac{{\text{TP}}_{i}}{{\text{TP}}_{i}+{\text{FN}}_{i}}$$

Equation 12 is the F1 score of the classification of Class *i*, which is the harmonic mean of the precision and recall rate.

(12) $$\text{F}\,1_{i}=\frac{2\cdot {\text{Precision}}_{i}\cdot {\text{Recall}}_{i}}{{\text{Precision}}_{i}+{\text{Recall}}_{i}}$$

Equations 13–15 are the macro average calculation formulae of the precision, recall rate and F1 score, which are obtained as the arithmetic average of various components.

(13) $${\text{Precision}}_{\text{macro}}=\frac{\sum_{i=1}^{L}{\text{Precision}}_{i}}{\left|L\right|}$$

(14) $${\text{Recall}}_{\text{macro}}=\frac{\sum_{i=1}^{L}{\text{Recall}}_{i}}{\left|L\right|}$$

(15) $$\text{Macro}\,\text{F}\,1=\frac{2\cdot {\text{Precision}}_{\text{macro}}\cdot {\text{Recall}}_{\text{macro}}}{{\text{Precision}}_{\text{macro}}+{\text{Recall}}_{\text{macro}}}$$

Equations 16–18 are the weighted average calculation formulae of the precision, recall rate and F1 score, which represent the weighted coefficients of a certain class in the total sample.

(16) $${\text{Precision}}_{\text{macro}}=\frac{\sum_{i=1}^{L}{\text{Precision}}_{i}\times w_{i}}{\left|L\right|}$$

(17) $${\text{Recall}}_{\text{macro}}=\frac{\sum_{i=1}^{L}{\text{Recall}}_{i}\times w_{i}}{\left|L\right|}$$

(18) $$\text{Macro}\,\text{weighted}\,\text{F}\,1=\frac{2\cdot {\text{Precision}}_{\text{macro}}\cdot {\text{Recall}}_{\text{macro}}}{{\text{Precision}}_{\text{macro}}+{\text{Recall}}_{\text{macro}}}$$

## Experimental Verification

### Experimental Verification of Facial Emotion Recognition Algorithm

#### Data Selection

In order to evaluate the performance of the proposed model, an evaluation experiment was designed. In the experiment, the CK+ data set of face emotion recognition was used. This data set has been widely used in experiments to evaluate the performance of facial emotion recognition algorithms, and consists of 593 video clips collected from 123 subjects of various races. Among them, 327 samples were marked with seven emotional labels: anger, contempt, disgust, fear, happy, sad and surprise. According to the recommendation of the data set, three frames from the middle of each video clip were intercepted as samples in our experiment to form a data set. This dataset is available online^1^.

As the number of data in the CK+ data set is limited, to enhance the generalization capacity of the model, the samples were rotated and balanced, and finally the data set was expanded to 16,890 samples.

#### Experimental Process

In the experiment, the data set to be tested was divided into a training set, a test set and a verification set by a ratio of 7:2:1, the data were randomly divided to ensure that the experimental results are reasonable and effective. In order to make the input samples compatible with the model, the images in the dataset were transformed into 8–8 bit gray images in 48 × 48 format prior to the experiment, and the label values were digitally encoded: 0–anger, 1–contempt, 2–disgust, 3–fear, 4–happy, 5–sad, and 6–surprise. The model was written in Python and trained with RTX-2070 graphics card. The number of training rounds were set to 500 rounds, but the training would be terminated if the loss function of verification set exceeded 50 rounds in the callback function, and the actual training rounds were about 60.

**FIGURE 8:**
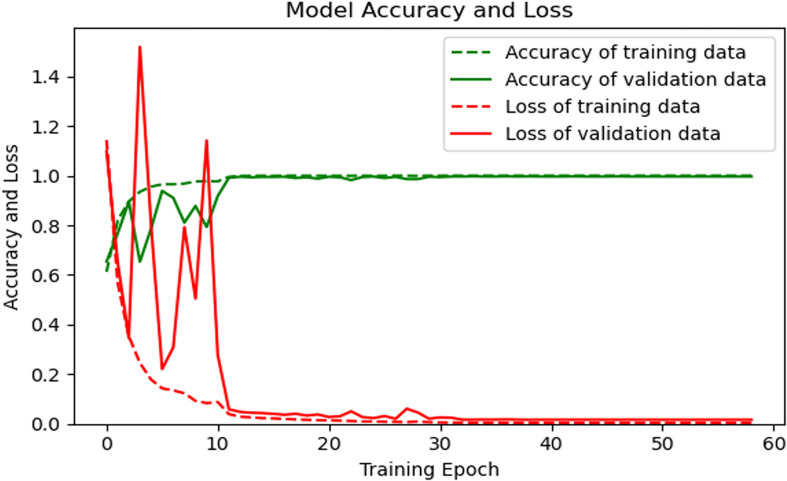
Loss and accuracy of the model on the training set and the verification set.

The loss function and accuracy curve of the training set and the verification set are shown in Figure 8.

As shown in Figure 8, after 20 rounds of training, the loss function curves and accuracy curves of the training set and the verification set tend to flatten out, and there is no obvious decline. After 50 training rounds, the accuracy of the training set and verification set has reached more than 99%, which proves the good performance of our proposed algorithm.

### Experimental Verification of Multi-Modal Physiological Signal-Based Stress Identification Algorithm

#### Data Selection and Preprocessing

In order to verify the effect of the proposed model, the published data set of multi-modal physiological stress identification (Birjandtalab et al., 2016) by the University of Texas was employed to test the model. This data set has collected five kinds of physiological signals from 20 college students (16 males and four females): triaxial acceleration, body temperature, galvanic skin response (GSR), Saturation of Peripheral Oxygen (SPO_2_) and heart rate, and in a period of time, a series of external environmental stimuli were applied to the subjects to direct them into four psychological stress states: physiological stress, cognitive stress, emotional stress and relaxed state. The relaxed state, as described in the work (Birjandtalab et al., 2016), was controlled to stay only within the first time period to make the quantity of data in each class of state more balanced. In addition, in the dataset, the sampling frequency of heart rate and blood oxygen signal in this data set is 1 HZ, while the sampling frequency of other physiological signals is 8 HZ. To make the sampling frequency of each physiological signal consistent, other physiological signals except the heart rate and the blood oxygen were down-sampled to a frequency of 1 HZ. After the above operations, four kinds of labels of physiological signals from 20 samples at 29,582 time points were obtained from the data set. In order to meet the requirements of the model input, the data was extracted by slices with 5 s as a unit. Label values were digitally coded beforehand: 0–relaxation, 1–physiological stress, 2–cognitive stress, and 3–emotional stress. This dataset is available online^2^.

#### Experimental Process

In the experimental, the data to be tested were divided into a training set and a test set by a ratio of 19:1; then, the verification set was separated from the training set by a ratio of 9:1. The data were randomly divided into different sets to ensure that the experimental results were reasonable and effective. The model was written in Python language, and RTX-2070 graphics card was used in the training process. The number of training rounds was set to 300, and the training would be terminated if the loss function of verification set exceeded 60 rounds in the callback function, and the actual training rounds were about 110 rounds. Figure 10 shows the loss and accuracy curves of the model on the training set and the verification set, and Figure 11 shows the confusion matrix on the test set.

As Figure 10 shows, the model achieves a good fitting effect, with an accuracy of 99.3% on the training set and 96.2% on the verification set, without overfitting. After 40 rounds of training, the loss and accuracy curves flatten out. As Figure 11 shows, cognitive stress marks the highest classification accuracy among all stress states.

## Discussion

### Discussion of of Facial Emotion Recognition Algorithm Experimental Results

Furthermore, the confusion matrix obtained by using the test set is shown in Figure 12.

As Figure 12 shows, the model also achieves high accuracy on the test set. Table 1 shows the specific results on the test set.

**TABLE 1:** Specific evaluation results on the test set.

Class	Precision	Recall	F1-score
Angry	0.99	0.99	0.99
Contempt	0.99	0.98	0.98
Disgust	0.99	0.99	0.99
Fear	0.99	0.99	0.99
Happy	0.99	0.99	0.99
Sad	0.97	0.99	0.98
Surprise	0.99	0.99	0.99
Macro average	0.99	0.99	0.99
Weighted mean	0.99	0.99	0.99

As Table 1 reveals, the results of the test set have reached an ideal range in terms of accuracy, F1 score and recall rate. Among them, the model performs best in classification of the emotions of anger and surprise. The accuracy of the test set is 99%, and the weighted F1 score is 0.99.

Comparisons between our model and previous works are shown in Table 2. As it suggests, our model gain an advantage over the previous advanced methods, thus rationalizing the application of the improved algorithm of mini_Xception in face emotion recognition and classification. Besides, because the mini_Xception model has a small scale of parameters and is applicable to low-performance platforms, the proposed improved algorithm can be well applied to the system, providing support for the facial emotion recognition function. The future research work can make breakthroughs in these aspects: (1) The parameter scale of feature extraction and the running time of the algorithm can be reduced to improve the efficiency of the system. (2) The model can be further improved and applied to continuous emotion recognition to enhance the universality of the model. (3) The performance of the model on emotion recognition accuracy under the condition of partial occlusion of the face can be tested and improved to enhance the generalization capacity of the algorithm.

**TABLE 2:** Comparison of performance on the dataset between different algorithms.

Source	Method	Accuracy	F1-score
Lopes et al. (2017)	7-Layers CNN	0.99	0.98
Lu et al. (2016)	LeNet-5	0.84	0.8456
Yong et al. (2018)	5-Layers CNN	0.97	0.9645
Xi et al. (2020)	CCRNet	0.98	0.9801
Our model	mini_Xception+GLCM feature extraction	0.99	0.99

### Discussion of Multi-Modal Physiological Signal-Based Stress Identification Algorithm Experimental Results

**FIGURE 9:**
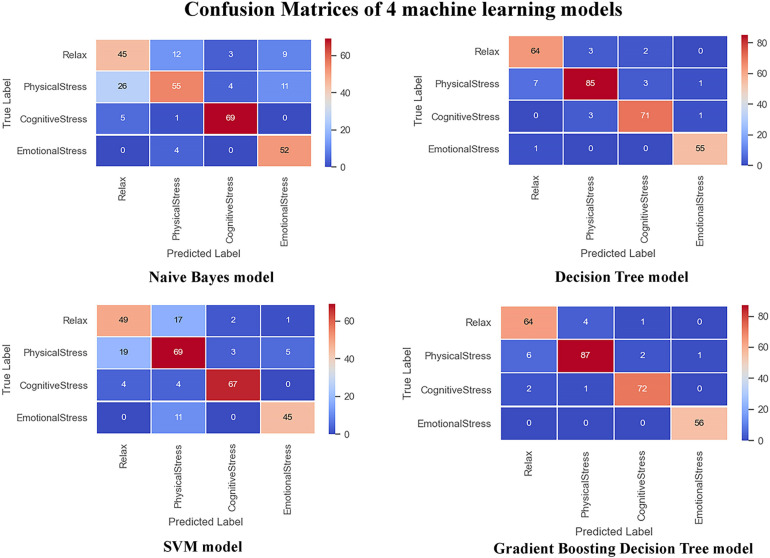
Confusion matrices of test set.

To verify the advantages of our model, it was compared with other machine learning models, including the support vector machine (SVM) model, decision tree model, classical Naive Bayes (NB) model, and gradient boosting decision tree (GBT) model. The form of data input remained the same on all the models for comparison. In addition, in order to better reflect the advantages of the proposed model, we compare with the Gaussian Mixture Model (GMM) used in the paper containing the original database and the Ensemble classifier based on statistical feature used in a research adopting the same database (Xin et al., 2019). Table 3 shows the comparison result between our model and the machine learning models as well as advanced methods from previous works, and Figure 9 show the performance of all the machine learning models that were compared.

**TABLE 3:** Model performance comparison.

Models	Accuracy	Weighted-recall	Weighted-F1 score
SVM	0.78	0.78	0.77
NB	0.75	0.75	0.75
GBT	0.94	0.94	0.94
Decision tree	0.93	0.93	0.93
This model	0.96	0.96	0.96
GMM	0.84	NA	NA
Ensemble classifier	0.94	0.94	NA

**FIGURE 10:**
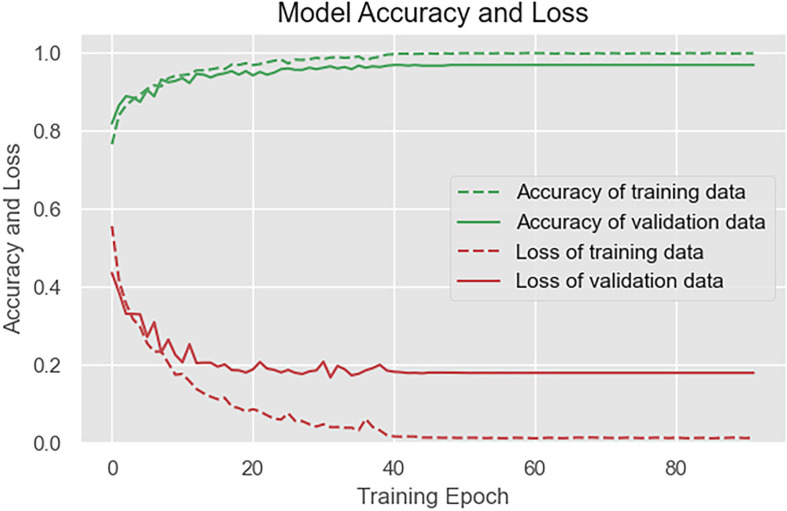
Loss and accuracy of the model on the training set and the verification set.

**FIGURE 11:**
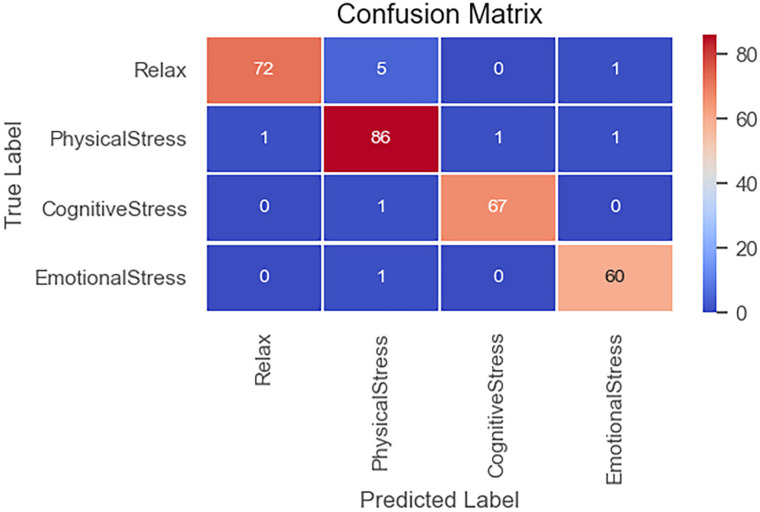
Confusion matrix of test set.

**FIGURE 12:**
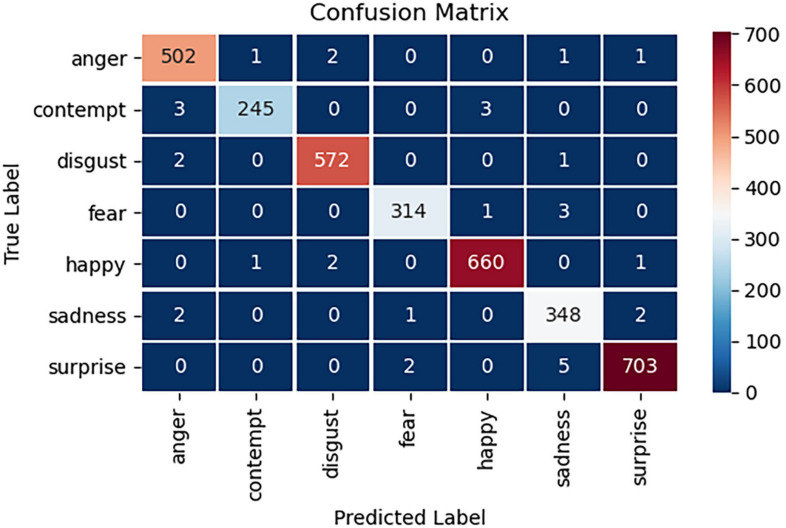
Confusion matrix on the test set.

As Table 3 shows, our model has absolute advantages over the classical Naive Bayes and SVM models, and it outperforms the random forest model and the gradient boosting decision-tree model in terms of accuracy. It could be also found that the proposed method outweights that of the previous works in terms of several crucial parameters. In addition, the variables input in the models are sliced and extracted, which contain more time features, so the model is expected to have better robustness in real-world scenarios. Nonetheless, as the model merges inputs from diverse channels, which increases the number of parameters. Convolutional layers dominate the computation complexity and consequently affects the latency and throughput (Jafari et al., 2018). In the future, more research work needs to be devoted to achieve a balance between the complexity and accuracy of the model.

### Discussion of Wearable Device System

Compared with previous work, the proposed wearable device has the following advantages: (1) a good balance is achieved between cost and functional effectiveness of the wearable device system; (2) the proposed device is portable and comfortable, thus making its application scenarios more general; (3) the system pays attention to the influence of external environment information, providing the basis for the follow-up research; (4) the integrated system could monitor both users’ stress and facial emotions of people around, which is suitable for research on facial emotion recognition disorders; (5) an original multi-modal physiological signal-based stress identification algorithm as well as an improved facial emotion recognition algorithm is carefully designed for the system.

However, this research also has many shortcomings. First of all, because facial emotion recognition disorders are related to mental illness, it is necessary to analyze the specific situation of different types of users to avoid the following problems: the users may not want to wear this device, and significant differences among specific users in the physiological signals would result in reduced accuracy of the model. In the future, surveys will be performed on the users to improve the shape, appearance and size of the device to increase its appeal to users. Moreover, more data on different groups of people should be collected to improve the model’s performance. Secondly, collecting information about emotions of people around and the surrounding environment by cameras may incur privacy disputes. Thus, in the future, it is necessary to improve the rules concerning the use of the device and the collected information to conform to the law and protect the privacy of users. Finally, the mobile APP designed in our system can provide only the result feedback function. Thus, it is necessary to expand the functions of the app to monitoring and analysis of the user’s movements, automatic emergency alarming and the like to improve user experience.

## Conclusion

In the present work, an ear wearable device system was proposed. The system can analyze the user’s own stress state and recognize the facial emotions of people around the user. It will provide a solution to long-term supervision of patients with facial emotion recognition disorders. The contributions of this work are as follows: A new platform is proposed, which can be used to assist and study facial emotion recognition disorders. The system is expected to provide help for patients or potential sufferers of facial emotion recognition disorders. Specifically, it can collect information and keep track of the stress state of the user, the surrounding environment, the emotions of people whom users interact with through sensors and cameras to realize real-time monitoring of the user’s psychological stress and allow the user to identify emotions of people around. The system can also be used by hospitals to analyze the patients’ specific conditions and make corresponding treatment plans. Moreover, a novel multi-modal physiological signal-based stress identification algorithm and an improved facial emotion recognition algorithm are put forward in this work, and experimental results show that these two algorithms could well meet the functional requirements of the system.

## Data Availability Statement

The original contributions presented in the study are included in the article/supplementary material, further inquiries can be directed to the corresponding author.

## Author Contributions

As the project leader and the first author, ZL was responsible for project establishment and assignment, modeling, heart rate data, facial expression data collection and analysis, and paper writing. YG was responsible for appearance design, experimental data processing, and paper revision. XC was responsible for hardware module design, experimental data processing, and paper revision. WL was responsible for communications and APP interface design. All authors contributed to the article and approved the submitted version.

## Conflict of Interest

The authors declare that the research was conducted in the absence of any commercial or financial relationships that could be construed as a potential conflict of interest.

## Footnotes

1 http://www.pitt.edu/ẽmotion/ck-spread.htm

2 https://physionet.org/content/noneeg/1.0.0/
